# JNK pathway activation is able to synchronize neuronal death and glial phagocytosis in *Drosophila*

**DOI:** 10.1038/cddis.2015.27

**Published:** 2015-02-19

**Authors:** J Shklover, K Mishnaevski, F Levy-Adam, E Kurant

**Affiliations:** 1 Department of Genetics and Developmental Biology, Faculty of Medicine, The Rappaport Family Institute for Research in the Medical Sciences, Technion—Israel Institute of Technology, Haifa 31096, Israel

## Abstract

Glial phagocytosis of superfluous neurons and damaged or aberrant neuronal material is crucial for normal development and maintenance of the CNS. However, the molecular mechanisms underlying the relationship between neuronal death and glial phagocytosis are poorly understood. We describe a novel mechanism that is able to synchronize neuronal cell death and glial phagocytosis of dying neurons in the *Drosophila* embryonic CNS. This mechanism involves c-Jun N-terminal kinase (JNK) signaling, which is required for developmental apoptosis of specific neurons during embryogenesis. We demonstrate that the dJNK pathway gain-of-function in neurons leads to dJNK signaling in glia, which results in upregulation of glial phagocytosis. Importantly, this promotion of phagocytosis is not mediated by upregulation of the glial phagocytic receptors SIMU and DRPR, but by increasing glial capacity to degrade apoptotic particles inside phagosomes. The proposed mechanism may be important for removal of damaged neurons in the developing and mature CNS.

During normal development of the central nervous system (CNS) a large number of neurons die through apoptosis^1, 2, 3, 4^ and are efficiently removed by phagocytic glia.^2, 5^ In the developing and adult CNS, different types of damage may occur, leading to neuronal injury. In these situations glia become reactive, upregulate their phagocytic ability and remove neuronal debris from the CNS.^6, 7, 8, 9^ However, the molecular mechanisms underlying the marked changes in glia remain elusive.

*Drosophila* glia are highly homologous functionally and molecularly to their mammalian counterparts.^10, 11, 12^ During embryogenesis, glial phagocytosis is determined by developmentally regulated expression of the phagocytic receptors Six Microns Under (SIMU) and Draper (DRPR), which does not depend on apoptosis.^13, 14, 15^ However, it is not clear how embryonic glia perform when neural damage occurs.

Recently, it has been shown that adult *Drosophila* glia become reactive in response to axotomy through *Drosophila* c-Jun N-terminal kinase (dJNK)–mediated activation of the phagocytic receptor DRPR.^9^ The *Drosophila* genome contains only a single JNK homolog, *basket* (*bsk*), as compared with *jnk1, jnk2* and *jnk3* genes in mammals.^16, 17^ As in mammals, activation of the JNK pathway in *Drosophila* initiates target gene transcription through phosphorylation-regulated activation of the AP-1s dJUN/JRA and dFOS/KAY. BSK's upstream JNK kinases are dMkk4 and Hemipterous (HEP) (Figure 1h).

dJNK signaling is essential for morphogenetic processes, wound healing, response to pathogens, tumor development, compensatory cell proliferation and apoptosis.^18^ In mammals, *jnk1/jnk2*-double knockout mice exhibit abnormal development of the embryonic brain, likely through inhibition of apoptosis,^19^ but there is little data about the role of the JNK pathway during *Drosophila* CNS development.^20^

Here, we show that the dJNK pathway is able to connect between neuronal death and upregulation of glial phagocytosis during *Drosophila* embryogenesis. Although the physiologically relevant role of this phenomenon is not yet clear, our model provides a potential mechanistic basis for connecting these two processes. We demonstrate that the dJNK pathway is normally involved in developmental neuronal apoptosis, but not in glial phagocytosis of apoptotic neurons. However, gain-of-function of dJNK signaling in embryonic neurons induces dJNK pathway activation in glia, which promotes glial phagocytosis of apoptotic cells. Importantly, this upregulation of phagocytosis is not accompanied by increased expression levels of the glial phagocytic receptors SIMU and DRPR or elevated engulfment ability of phagocytic glia, but it promotes degradation of engulfed apoptotic particles. Our work recognizes dJNK signaling as a possible mechanism of upregulation of glial phagocytosis through enhancement of glial capacity to degrade apoptotic particles.

## Results

### The dJNK pathway is active in the embryonic CNS in specific neurons and is involved in developmental neuronal apoptosis

**Figure 1:**
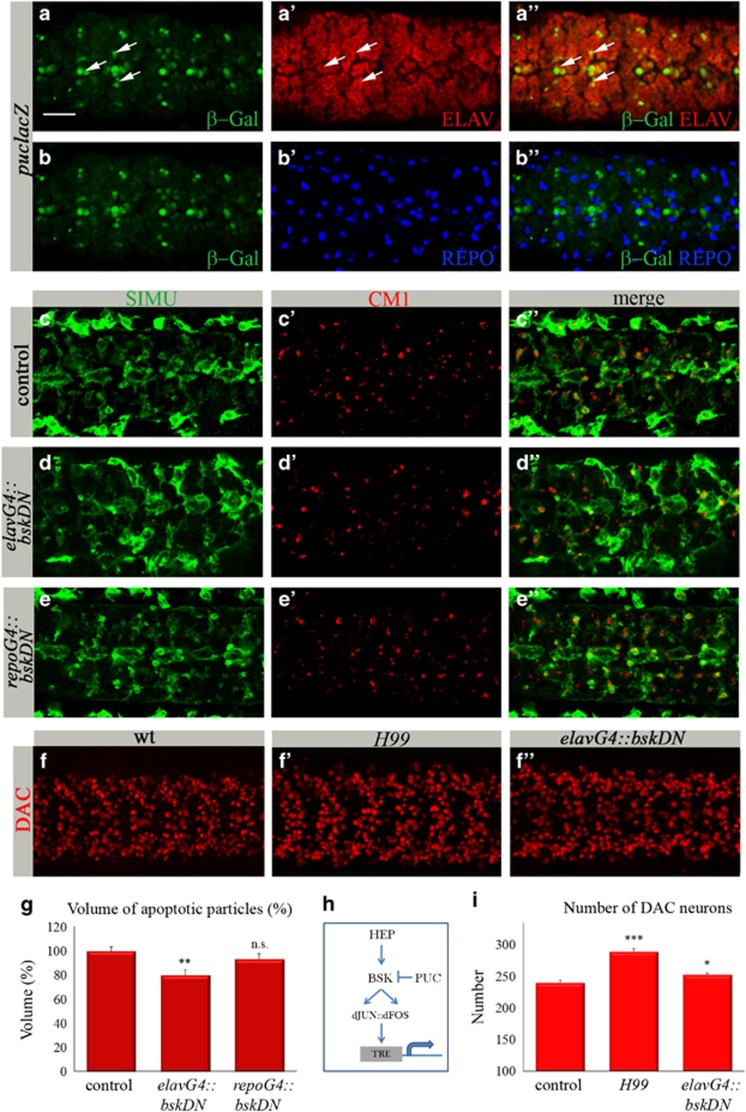
The dJNK pathway is active in embryonic neurons and is required for neuronal apoptosis. (**a**–**f″**) Projections from confocal stacks of the embryonic CNS at stage 16; ventral view. Bar 20 *μ*m. (**a**–**b″**) Embryos expressing *puclacZ* reporter stained with anti-*β*-Gal (green), anti-ELAV (red) and anti-REPO (blue) antibodies. Some neurons exhibiting *puclacZ* expression are marked with arrows. (**c**–**e″**) Control embryos and embryos expressing *bskDN* in neurons (*elavGal4*::*bskDNK53R*) or glia (*repoGal4*::*bskDNK53R*) are labeled with anti-activated caspase 3 (CM1, red) and glia (anti-SIMU, green). (**f**–**f″**) Neurons labeled with anti-DAC (red) in control, *H99,* and *elavGal4::bskDNK53R* embryos. (**g** and **i**) Columns represent mean total volume of apoptotic particles (**g**) or DAC-positive neurons (**i**) within confocal stacks of the CNS,±S.E.M., *n*=7–12; asterisks indicate statistical significance *versus* control, as determined by the Student's *t*-test, ***P<0.0001, **P<0.002, *P<0.04, NS (not significant) *P*>0.05. (**h**) Schematic representation of dJNK signaling to the nucleus to induce gene expression

Massive neuronal apoptosis takes place in the *Drosophila* CNS during late stages of embryogenesis.^13, 21^ As the dJNK pathway has been shown to induce ectopic apoptosis in imaginal epithelia of eye and wing disks^22, 23^ and *bsk* RNA is specifically expressed in the late embryonic CNS,^16^ we examined whether the dJNK pathway is active in the embryonic CNS. We followed expression of a *lacZ* enhancer trap in the gene *puckered* (*puclacZ* reporter), which is a target of the dJNK pathway and also inhibits it by a feedback loop.^24^ To examine the embryonic CNS of *puclacZ* transgenic embryos, we used differential labeling of neurons and glia by specific antibodies against ELAV (Figure 1a′) and REPO (Reverse Polarity; Figure 1b′), respectively. In these embryos, we detected colocalization of *β*-Gal (Figures 1a and b) with ELAV (Figure 1a′′), but not with REPO (Figure 1b′′) staining, suggesting that during late embryogenesis the dJNK pathway is active in specific neurons but not in glia.

To test the role of dJNK signaling in neuronal apoptosis, we knocked down BSK function by expressing its dominant-negative (DN) form, *bskDN,* specifically in neurons using the *elavGal4* driver. To evaluate the levels of apoptosis in the CNS, we used an anti-activated caspase 3 antibody (CM1) for labeling apoptotic particles (Figures 1c′). Specific impairment of BSK function in post-mitotic neurons led to a small but significant reduction in apoptotic cell volume as compared with control embryos (Figure 1g), suggesting that inhibition of dJNK signaling prevented caspase activation and apoptosis of certain neurons. To corroborate this result, we assessed neuronal death by quantifying neurons in mutant and control embryos. As using a pan-neuronal marker anti-ELAV (Figure 1a′) is not feasible for counting, we used anti-Dachshund (DAC), which specifically labels a subset of neurons, whose number is easier to count (Figures 1f and i). We first verified that DAC-positive neurons die during normal embryogenesis, using the anti-DAC staining of *H99* embryos (Figure 1f′). *H99* embryos are *hid, reaper and grim* deficient and lack caspase activation.^13, 25^ Counting the DAC-positive neurons in *H99* mutant embryos indicated a significant increase in their number compared with wild-type (wt) embryos (Figure 1i), indicating that these neurons normally die through apoptosis. Importantly, expressing *bskDN* in glia *(repoGal4* driver), did not affect the volume of apoptotic particles and the shape of glial cells (Figures 1e–e′′ and g) as compared with control embryos (Figures 1c–c′′ and g). These results indicate that the dJNK pathway does not function in embryonic glia, which is consistent with the lack of *puclacZ* reporter expression in glial cells (Figure 1b′′).

### Gain-of-function of dJNK signaling leads to upregulation of glial phagocytosis

**Figure 2:**
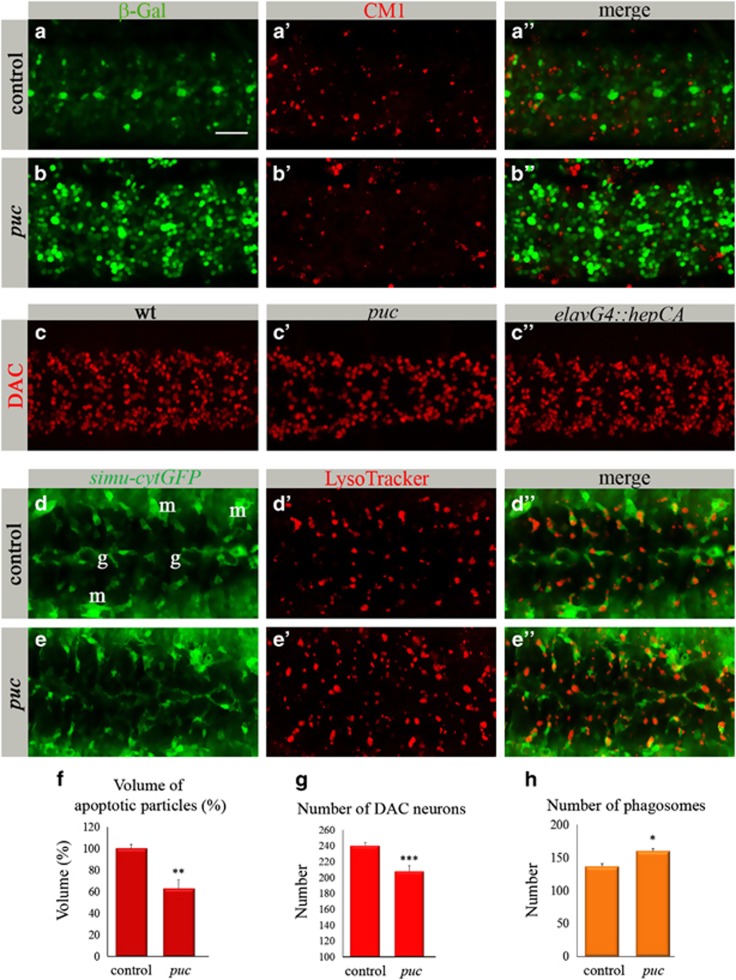
Gain-of-function of the dJNK pathway reduces the volume of apoptotic particles in the CNS. (**a**–**c″**) Projections from confocal stacks of the embryonic CNS at stage 16; ventral view. Bar 20 *μ*m. (**a**–**b″**) Embryos expressing *puclacZ* reporter stained with anti-*β*-Gal (green) and CM1 (red) antibodies. (**a**–**a″**) *puclacZ* heterozygous embryos as a control. (**b**–**b″**) *puclacZ* homozygous mutant embryos. (**c**–**c″**) Neurons labeled with anti-DAC (red). (**d**–**e″**) Projections from confocal stacks of the embryonic CNS of stage-16 live embryos; ventral view. *simu-cytGFP* reporter marks glia (g) and macrophages (m) in green. Phagosomes are marked with LysoTracker (red). (**f**) Volume of apoptotic particles within CNS sections. Columns represent mean total volume of apoptotic particles within confocal stacks of the CNS, ±S.E.M., *n*=7–8; asterisks indicate statistical significance *versus* control, as determined by the Student's *t*-test, **P<0.003. (**g**) Quantification of DAC-positive neurons. To count DAC-positive neurons, confocal stacks of the whole CNS (15 sections; total 22.5 *μ*m) were acquired. Columns represent mean total number of DAC-positive neurons in three segments within confocal stacks of the CNS, ±S.E.M., *n*=8–15; asterisks indicate statistical significance *versus* control, as determined by the Student's *t*-test, ***P<0.0001. (**h**) Number of phagosomes within CNS sections. Columns represent mean total number of phagosomes within confocal stacks of the CNS, ±S.E.M., *n*=6–7; asterisks indicate statistical significance *versus* control, as determined by the Student's *t*-test, *P<0.04

Given that dJNK signaling is involved in apoptosis of certain neurons, we expected its gain-of-function would lead to higher apoptosis rates in the CNS. Therefore, we tested levels of apoptosis in *P{A92}puc*^*E69*^ *(puc)* mutant embryos, in which the dJNK pathway is overactivated because of the removal of Puc inhibition. *puc* mutant embryos exhibited a large volume of apoptotic particles outside the CNS (Supplementary Figure S1), resulting probably from the excessive dJNK pathway activity at earlier embryogenesis. Surprisingly, a significantly lower volume of apoptotic particles was detected inside the CNS of these embryos compared with control heterozygous embryos (Figures 2a′).

A reduction in the volume of apoptotic particles would be detected when less apoptosis occurs or alternatively, when phagocytosis of apoptotic particles is more efficient, leading to their faster degradation inside phagocytes and therefore to their lower amount. To assess neuronal loss, we quantified DAC-positive neurons in *puc* mutant and control embryos and detected a significantly lower number in the mutants (Figures 2c, c′ and g). To test whether the reduced number of DAC-positive neurons is a result of apoptosis or of their diminished production, we counted them in mutant embryos at early stage 14, before a pick of neuronal developmental apoptosis occurs and after the beginning of *puc* expression in the CNS.^21^ The same number of DAC-positive neurons was detected in mutant and control embryos (Supplementary Figure S2), indicating no defects in their production. Moreover, to test a possible change in cell fate of these neurons in the mutant, we quantified CUT- and EVE-positive neurons, as well as REPO-positive glial cells in stage-16 embryos. None of these cell types showed any increase in their number compared with wt (Supplementary Figure S3; Figure 4e), suggesting DAC-positive neurons in *puc* mutant embryos are rather eliminated through cell death. Therefore, the decreased volume of apoptotic particles observed in mutant embryos indicates that neurons may disappear through caspase-independent cell death or accelerated glial phagocytosis.

To evaluate the level of glial phagocytosis in *puc* mutants, we used LysoTracker (LT) labeling of phagosomal activity in glial cells (Figures 2d–e′′). Wt embryonic glia are highly phagocytic during late embryogenesis, as reflected by LT-positive phagosomes inside GFP-labeled glial cells (Figure 2d′′). Quantification of the data revealed significantly higher number of phagosomes in the *puc* mutant than in the control CNS (Figures 2d′), supporting the assumption of upregulated phagocytic activity. These data imply that in *puc* embryos, the higher level of neuronal death is accompanied by upregulated glial phagocytosis.

### Overactivation of dJNK signaling in neurons leads to dJNK signaling in glia

**Figure 3:**
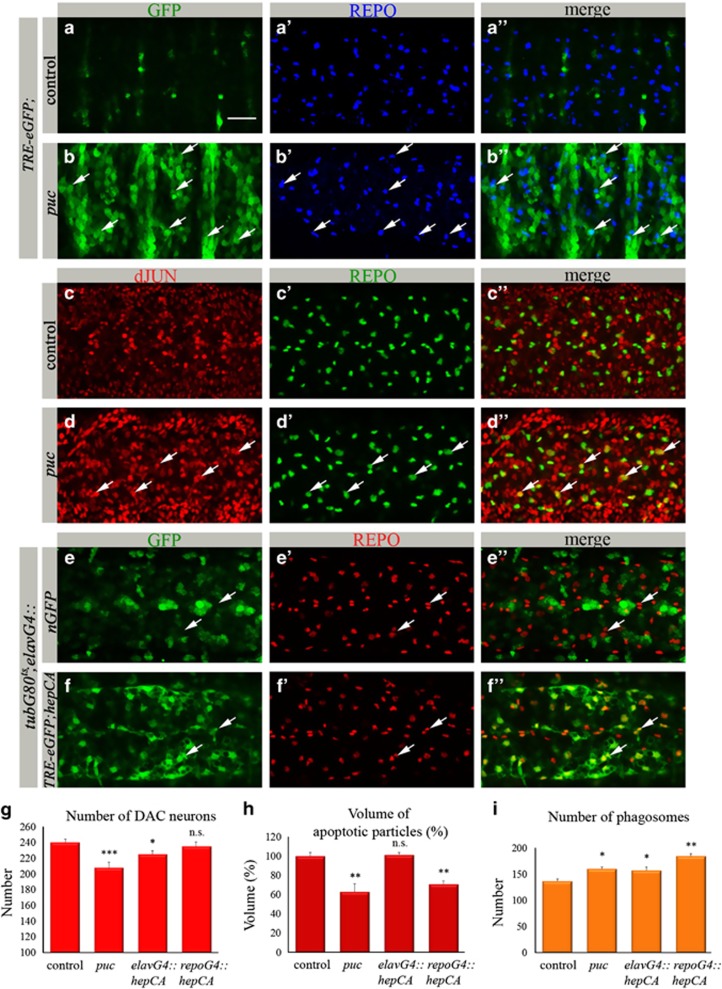
Gain-of-function of dJNK signaling in neurons upregulates glial phagocytosis. (**a**–**f″**) Projections from confocal stacks of the embryonic CNS at stage 16; ventral view. Bar 20 *μ*m. (**a**–**b″**) *TRE-eGFP* reporter (in green) depicts dJNK pathway activation. REPO-positive glial nuclei are marked in blue. (**a**–**a″**) *repoGal4* control. (**b**–**b″**) *puc*^*E69*^ mutant embryo. Arrows depict glial cells expressing *TRE-eGFP* reporter. (**c**–**d″**) anti-dJUN staining in red and anti-REPO in green. (**c**–**c″**) *repoGal4* control. (**d**–**d″**) *puc*^*E69*^ mutant embryo. Arrows mark glial cells expressing dJUN. (**e**–**f″**) Glia in red (anti-REPO). (**e**–**e″**) *tubGal80*^*ts*^*; elavGal4::nucGFP* embryos. No nuclear GFP expression in glia (arrows). (**f**–**f″**) *tubGal80*^*ts*^*; elavGal4::TRE-eGFP;hepCA* embryos showing *TRE-eGFP* expression in glial cells (arrows). (**g**) Quantification of DAC-positive neurons. Columns represent mean total number of DAC-positive neurons within confocal stacks of the CNS, ±S.E.M., *n*=6–12. (**h**) Volume of apoptotiPlc particles within CNS sections. Columns represent mean total volume of apoptotic particles within confocal stacks of the CNS, ±S.E.M., *n*=7–13. (**i**) Number of phagosomes within CNS sections. Columns represent mean total number of phagosomes within confocal stacks of the CNS, ±S.E.M., *n*=6–7. (**g**–**i**) Asterisks indicate statistical significance *versus* control, as determined by the Student's *t*-test, ***P<0.0001, **P<0.003, *P<0.04, NS (not significant) *P*>0.05

To further understand the molecular basis of increased glial phagocytosis in *puc* mutants, we introduced a reporter for dJNK activity, *TRE-eGFP* that contains *Drosophila* AP-1-binding sites fused to the *eGFP* gene^9, 26^ (Figure 1h). We observed a robust neuronal expression of *TRE-eGFP* in *puc* mutant embryos (Figures 3b and b′′); however, not all of these neurons died as suggested by the quantification of DAC-positive neurons (Figure 2g). These data propose that dJNK gain-of-function alone is not sufficient to kill embryonic neurons.

Interestingly, in addition to its neuronal expression, *TRE-eGFP* was detected in some glial cells in *puc* embryos (Figures 3b–b′′), indicating that dJNK signaling was activated in glia as well. We confirmed dJNK pathway activation in glia by immunostaining of *puc* mutants with an anti-dJUN antibody. A significantly higher signal of dJUN was detected in mutant neurons and glia as compared with control embryos (Figures 3c, c′′), again demonstrating dJNK pathway activation also in glia.

To test a possible connection between dJNK signaling in neurons and glia as suggested in *puc* mutants, we sought to test whether specific overactivation of the dJNK pathway in neurons would lead to dJNK signaling in glia. To activate the pathway in neurons, we expressed the constitutively active HEP (*hepCA)* with the *elavGal4* driver. It has been previously shown that *elavGal4* is transiently expressed in embryonic glia.^27^ In order to prevent dJNK pathway activation directly in glia, we used *tubGal80* repression of the *elavGal4* driver until embryonic stage 14, when *elavGal4* expression in glia is terminated,^27^ and tested its expression with nuclear GFP. No GFP expression in glia was detected in these embryos, indicating that *elavGal4* was solely expressed in neurons (Figures 3e–e′′). Using the same time courses we expressed *hepCA* in neurons and followed *TRE-eGFP* expression. A strong GFP expression was detected in glial cells of these embryos (Figures 3f–f′′), suggesting dJNK signaling in glia as the result of dJNK pathway overactivation specifically in neurons. In these embryos, a significantly lower number of DAC-positive neurons was detected (Figure 3g), but no increase in the volume of apoptotic particles (Figure 3h). Importantly, a significantly elevated number of LT-positive phagosomes (Figure 3i) indicated upregulated phagocytic activity of glial cells. These data suggest that overactivation of the dJNK pathway in neurons leads to dJNK signaling in glia, resulting in promotion of glial phagocytosis.

### Glial phagocytosis is upregulated via activation of dJNK signaling in glial cells

**Figure 4:**
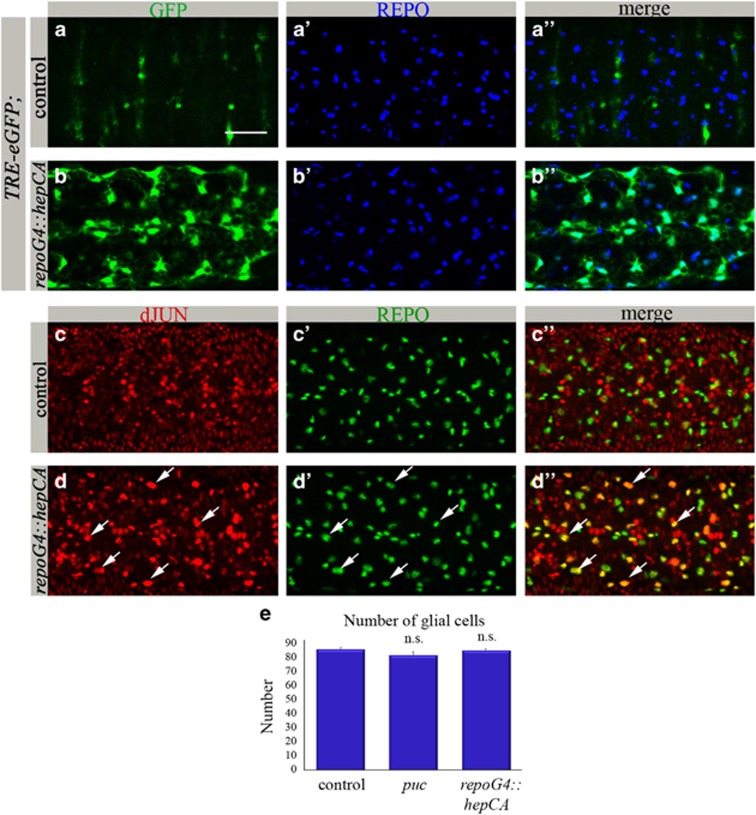
Activation of dJNK signaling in glial cells promotes glial phagocytosis. (**a**–**d″**) Projections from confocal stacks of the embryonic CNS at stage 16; ventral view. Bar 20 *μ*m. (**a**–**b″**) *TRE-eGFP* reporter (in green) depicts JNK pathway activation. REPO-positive glial nuclei are marked in blue. (**a**–**a″**) *repoGal4* control. (**b**–**b″**) *repoGal4::hepCA* mutant embryo. (**c**–**d″**) anti-dJUN staining in red and anti-REPO in green. (**c**–**c″**) *repoGal4* control. (**d**–**d″**) *repoGal4::hepCA* mutant embryo. Arrows mark glial cells expressing dJUN. (**e**) Number of REPO-positive nuclei within CNS sections. Columns represent mean total number of glial nuclei within confocal stacks of the CNS, ±S.E.M., *n*=5–8; NS (not significant) *P*>0.05, as determined by the Student's *t*-test. There is no significant difference in number of glial nuclei between *repoGal4::hepCA*, *puc*^*E69*^ mutant and control embryos

To gain deeper insight into dJNK signaling in glia, we examined embryos ectopically expressing the constitutively active HEP specifically in glia *(repoGal4::hepCA*). In these embryos high levels of *TRE-eGFP* were detected exclusively in glia (Figures 4b–b′′). dJNK pathway activation was also demonstrated by much stronger dJUN in glia as compared with control embryos (Figures 4c–d′′). Strikingly, there was a significantly lower amount of apoptotic particles in the CNS of these embryos as compared with the control (Figure 3h). However, quantification of DAC-labeled neurons showed no significant difference between embryos containing ectopic dJNK signaling in glia and control embryos (Figure 3g), indicating that dJNK pathway activation in glia does not affect neuronal death but rather suggests faster elimination of already dying neurons. This conclusion was confirmed by a significantly higher number of phagosomes in the embryonic glia where the dJNK pathway was activated, than in the control CNS (Figure 3i), demonstrating that dJNK signaling promotes glial phagocytic activity. No significant difference in the amount of REPO-positive nuclei was perceived between mutant and control embryos (Figures 4a′), indicating that dJNK signaling in glia does not affect glial number.

### dJNK signaling promotes glial phagocytosis downstream of SIMU and DRPR

**Figure 5:**
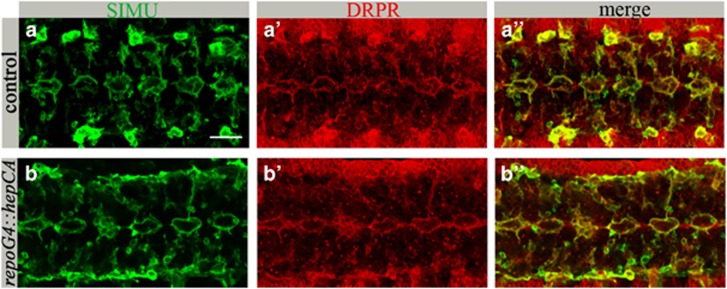
dJNK activation in glia does not affect SIMU or DRPR expression. (**a**–**b″**) Projections from confocal stacks of the CNS at embryonic stage 16, ventral view. Bar 20 *μ*m. (**a**–**a″**) *repoGal4* control. (**b**–**b″**) *repoGal4::hepCA* embryo showing no change in anti-SIMU (green) or anti-DRPR (red) staining

To better understand the molecular mechanisms underlying dJNK-mediated upregulation of glial phagocytosis, we first examined the protein levels of SIMU and DRPR receptors in glia exhibiting activated dJNK signaling. Similar levels and distribution of SIMU and DRPR were observed on glial membranes of control and mutant embryos (Figures 5a, a′). These data are in agreement with our previous results, where we demonstrated that embryonic glia managed to engulf the increased number of apoptotic particles without changing the expression levels of SIMU and DRPR.^15^

**Figure 6:**
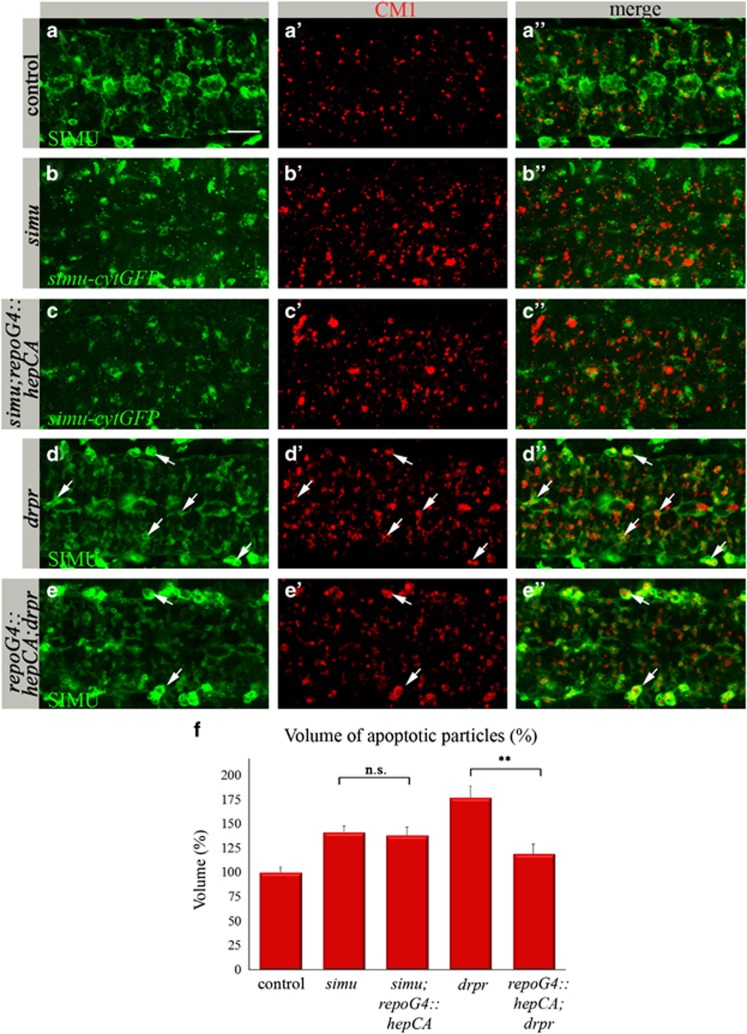
dJNK signaling in glia does not rescue the *simu* phenotype, whereas it substantially rescues the *drpr* phenotype. (**a**–**e″**) Projections from confocal stacks of the CNS at embryonic stage 16, ventral view. Apoptotic cells in red (CM1), whereas glia (in green) are labeled with different markers. Bar 20 *μ*m. In control *repoGal4* embryo (**a**–**a″**) apoptotic particles are mostly inside anti-SIMU-labeled glia. In the *simu* mutant (**b**–**b″**), many apoptotic particles are outside glia labeled with *simu-cytGFP*. (**d**–**d″**) In *the drpr* mutant, apoptotic particles accumulate inside glia and macrophages (arrows). (**c**–**c″** and **e**–**e″**) Rescue experiments of *simu* (**c**–**c″**) or *drpr* (**e**–**e″**) mutants by dJNK signaling in glia (*repoGal4::hepCA)*. No rescue is detected in the *simu* mutant (**c**–**c″** and **f**) but substantial rescue is detected in the *drpr* mutant (**e**–**e″** and **f**). Many apoptotic particles accumulate in macrophages outside the CNS (arrows) but not in glia (**e″**). (**f**) Quantification of phenotypic rescue of *simu* and *drpr* mutants by *repoGal4::hepCA*. Columns represent mean total volume of apoptotic particles within confocal stacks of the CNS, ±S.E.M., *n*=5–9; asterisks indicate statistical significance *versus simu* and *drpr* mutants, as determined by the Student's *t*-test, **P<0.002, NS (not significant) *P*>0.05

To examine whether engulfment and/or degradation of apoptotic particles is enhanced by activation of the dJNK pathway irrespective of *simu* and *drpr* activation, we evaluated the ability of glial dJNK signaling to rescue *simu* and *drpr* mutant phenotypes. In the embryonic CNS, *simu* is required for recognition and engulfment of apoptotic neurons by glia, whereas *drpr* is mostly involved in their degradation inside glial cells.^13^ We activated dJNK signaling *s*pecifically in glia of *simu* (*simu;repoGal4::hepCA*) or *drpr* (*repoGal4::hepCA;drpr*) mutant embryos. *simu* mutant embryos exhibit a significantly higher volume of apoptotic particles in the CNS^13^ (Figures 6b′) compared with control embryos (Figures 6a′), and most of them are found outside glial cells labeled by *simu-cytGFP* reporter (Figure 6b′′).^13^ dJNK pathway activation specifically in glia of *simu* mutant embryos (Supplementary Figure S4) did not rescue the abnormal phagocytosis phenotype (Figures 6c′), suggesting that dJNK signaling is unable to overcome defects in recognition and engulfment of apoptotic particles.

**Figure 7:**
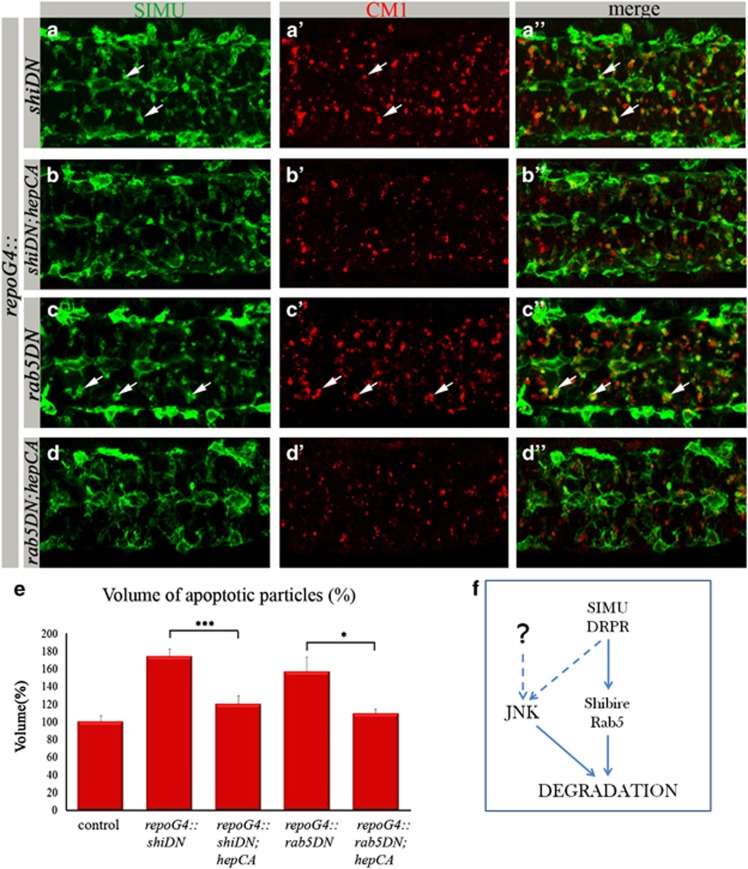
dJNK signaling in glia rescues shibire- and *rab5*- associated defects in apoptotic particle degradation. (**a**–**d″**) Projections from confocal stacks of the CNS at embryonic stage 16, ventral view; apoptotic cells are in red (CM1) and glia labeled with anti-SIMU in green. Bar 20 *μ*m. *repoGal4::shibireDN* (**a**–**a″**) and *repoGal4::rab5DN* (**c**–**c″**) embryos show an increased volume of apoptotic particles compared with control (Figures 6a′ and a″), which are found mostly inside SIMU-labeled glia (arrows). In rescue experiments with *repoGal4::hepCA* (**b**–**b″** and **d**–**d″**) a substantial rescue is detected. (**e**) Quantification of phenotypic rescue of shibire and *rab5* dominant-negative mutants by constitutively active HEP. Columns represent mean total volume of apoptotic particles within confocal stacks of the CNS, ±S.E.M., *n*=6–8; asterisks indicate statistical significance *versus repoGal4::shibireDN* and *repoGal4::rab5DN*, as determined by the Student's *t*-test, ***P<0.002, *P<0.03. (**f**) Schematic representation of dJNK signaling in glia downstream of SIMU and DRPR

However, we detected a substantial rescue of the *drpr* mutant phenotype (Figures 6d–d′′) by dJNK pathway activation specifically in glia, exhibiting many fewer apoptotic particles inside glial cells, whereas macrophages present outside the CNS display the strong *drpr* phenotype (Figures 6e–e′′). These data place the dJNK pathway activity downstream of SIMU and DRPR function in glial phagocytosis, likely in degradation of apoptotic particles inside glial cells (Figure 7f).

### dJNK signaling in glia rescues defects in degradation of apoptotic particles

To further assess how dJNK signaling in glia promotes phagocytosis, we tested components of the basic intracellular phagocytic machinery, which act downstream of *simu* and *drpr* in degradation of apoptotic particles. We tested *rab5* and the *Drosophila* homolog of *Dynamin*, *shibire*, both required for endosome–phagosome tethering and fusion.^28, 29, 30, 31, 32, 33, 34^ Specific expression of DN forms of shibire and *rab5* in glia (*repoGal4::shibireDN* and *repoGal4::rab5DN)* caused a strong phagocytosis phenotype, as evaluated by CM1 and anti-SIMU staining (Figures 7a′). These embryos exhibited large numbers of apoptotic particles in the CNS and, importantly, most of the particles were found inside glial cells, indicating their abnormal or delayed degradation. Strikingly, when we expressed the DN form of *shibire* together with *hepCA* specifically in glia (*repoGal4::shibireDN;hepCA*), we detected a significantly lower volume of apoptotic particles as compared with *the shibire* mutant alone, indicating a substantial rescue of the abnormal degradation phenotype (Figures 7b′). Similarly, when *hepCA* was expressed with the rab5DN form, a significant rescue of the mutant phenotype was observed as well with many fewer apoptotic corpses inside glia (Figures 7d′). These results suggest that dJNK signaling accelerates phagocytosis by stimulating more efficient degradation of engulfed particles (Figure 7f).

## Discussion

Glial phagocytosis has a critical role during *Drosophila* normal embryonic development, neuronal pruning and axonal degeneration.^8, 9, 13, 15, 35, 36, 37, 38, 39^ However, much less is known about the molecular mechanisms controlling phagocytic glia when damage occurs in the developing CNS. JNK signaling is one of the major stress response pathways, which often triggers apoptosis.^18, 40, 41, 42, 43^ The current work shows that dJNK signaling, which is active in certain embryonic neurons, normally dying through apoptosis, is able to synchronize excess neuronal death with upregulation of glial phagocytosis of dying neurons. This promotion of glial phagocytic ability does not affect levels of the phagocytic receptors SIMU and DRPR, but apparently upregulates the intracellular phagocytic machinery responsible for phagosome maturation and degradation of apoptotic particles (Figure 7f). We suggest that this ability of dJNK signaling to promote phagocytic removal of dying cells might be employed for efficient clearance of damaged or aberrant material during CNS development and injury.

In a normal adult CNS, there is almost no cell death and glial phagocytosis of dying neurons. However, it has been recently shown that after neuronal injury, *Drosophila* glia upregulate their basal ability to phagocytose through activation of the dJNK pathway, leading to elevation of DRPR levels.^9^ In contrast, during late embryogenesis, massive neuronal apoptosis takes place, and highly phagocytic glia efficiently clear the apoptotic neurons. At this stage, the phagocytic receptors SIMU and DRPR are highly expressed in glia and have a crucial role in engulfment and degradation of apoptotic cells.^13, 15^ We have recently demonstrated that embryonic glia are able to clear excess apoptotic particles without increasing SIMU and DRPR levels, and that their expression in the embryonic CNS is controlled by the glial developmental program.^15^ Here we show that this developmental program normally does not involve dJNK pathway activation. Moreover, we demonstrate that ectopic activation of dJNK signaling in embryonic glia does not affect the levels of SIMU and DRPR.

However, we discovered that overexpression of the activated dJNK pathway in embryonic neurons leads to dJNK-mediated upregulation of glial phagocytosis, suggesting that dJNK signaling prepares neighboring glia for removing damaged neuronal material. Importantly, neuronal caspase activation induced by head involution detective (HID) or Reaper did not activate the dJNK pathway in embryonic glia (Supplementary Figure S5), demonstrating that enhanced glial ability to phagocytose is not triggered by neuronal apoptosis *per se*. A similar mechanism was proposed in elimination of oncogenic cells in *Drosophila* imaginal epithelia, where dJNK signaling in tumor cells induced their dJNK-dependent phagocytosis by neighboring cells.^44^ In addition, JNK pathway activity has been detected in mammalian macrophages and epithelial cells during engulfment of apoptotic cells *in vitro*,^45, 46^ suggesting a conserved role for JNK signaling in phagocytosis.

Importantly, despite the robust gain-of-function of dJNK signaling in embryonic neurons, only a relatively small number of neurons died and were removed by phagocytic glia. This suggests that activation of the dJNK pathway alone is not sufficient to kill developing neurons, and other signals are probably required to cause neural cell death. The additional scenario might be repression of dJNK signaling in neurons by developmental regulators, similar to the Decapentaplegic-mediated protection of epithelial cells from dJNK-induced apoptosis during dorsal closure.^47^

As mentioned above, previous studies demonstrated that dJNK signaling in phagocytic cells upregulates expression of the phagocytic receptor DRPR.^9, 48^ Our data demonstrate that dJNK pathway activation in embryonic glia does not elevate the levels of SIMU and DRPR, suggesting that they are not a limiting factor for glial phagocytosis during embryogenesis. However, we demonstrate the ability of dJNK signaling to substantially rescue abnormal degradation of apoptotic particles inside glial cells downstream of DRPR function. These results argue that during late embryogenesis, dJNK signaling is able to upregulate glial phagocytosis through acceleration of the intracellular degradation machinery, and demonstrate for the first time the involvement of dJNK signaling in degradation of apoptotic particles.

The physiological or pathological conditions under which JNK signaling connects neuronal damage and upregulation of glial phagocytosis are currently unclear, and require additional studies. However, it is likely that when the trigger for dJNK signaling overactivation in embryonic neurons is identified it will work through a mechanism consistent with the data provided here. Various types of intrinsic and environmental stresses activate the JNK pathway in mammalian neurons, leading to neuronal death, such as UV irradiation, reactive oxygen species, DNA damage, heat shock, bacterial antigens, and inflammatory cytokines.^49^ In *Drosophila*, JNK signaling has been shown to have a critical role in elimination of aberrant cells during development.^18^ Moreover, in the *Drosophila* eye model of Alzheimer disease, JNK signaling was induced in neurons by accumulation of A*β*42 plaques, which contributed to A*β*42-mediated cell death.^50^ It would be intriguing to evaluate the state of glia in similar situations of neuronal death. More competent phagocytic glia could be an advantage under neuronal stress conditions in the developing and adult CNS.

## Materials and Methods

### Fly strains

The following fly strains were used in this work: *repoGal4* (B Jones), *elavGal4* (O Schuldiner), *UAScytGFP* (#1521; Bloomington), *UASnGFP* (#4775; Bloomington), *TRE-eGFP* (D Bohmann), *UAShid* and *UASreaper* (E Arama), *simu* and *simu-cytGFP*,^13^ *drpr* (M Freeman), *tubGal80*^*ts*^ (#7019; Bloomington), *UASrab5S43N* (#42703; Bloomington), *UASshiK44A* (#5811; Bloomington), *puclacZ=puc[E69]* (A Salzberg). *P{A92}puc*^*E69*^ mutant embryos comprise a *lacZ*-containing P-element inserted into the *puc* endogenous locus, which disrupts *puc* normal function.^51^ *UAShepCA* (#9306, #6406; Bloomington), *UASbskK53R=bskDN* (#9311; Bloomington). For neuron-specific expression of *elavGal4,* embryos were placed at 18 °C until early stage 14 and was then transferred to 29 °C for 4 h. For *hid* expression in larval neurons, we placed *elavGa4::hid; tubGal80*^*ts*^ progeny at 18 °C until the 2nd instar larval stage and then shifted them to 29 °C for 24 h.

### Immunohistochemistry and live imaging

Guinea pig anti-DRPR antibody was raised against a GST-tagged fragment comprising the last 220 amino acids of the DRPR protein. For immunohistochemistry, embryos were fixed and stained according to standard procedures. Guinea pig anti-SIMU^25^ and guinea pig anti-DRPR were used at a 1 : 5000 and 1 : 100 concentrations, respectively. Rabbit anti-activated caspase 3 (CM1, BD, Franklin Lakes, NJ, USA) and mouse anti-GFP (Roche, Mannheim, Germany) were used at 1 : 50 and 1 : 100 concentrations, respectively. Rabbit anti-dJUN was a gift from D Bohmann and used at 1 : 400 dilution. Mouse anti-REPO (1 : 20), anti-CUT (1 : 50), anti-EVE (1 : 100) and anti-DAC (1 : 5) were from Hybridoma bank (Iowa city, IA, USA). Fluorescent secondary antibodies Cy3/488/647 from Jackson ImmunoResearch (West Grove, PA, USA) were used at 1 : 200 dilutions. Glycerol solution (80%) was used as the imaging medium. All confocal images were acquired on a confocal microscope Zeiss (Aalen, Germany) LSM 700 using a Plan-Apochromat × 20/0.8 M27 lens. Image analysis was performed using Zeiss LSM 700 and Imaris (Bitplane, Zurich, Switzerland) software. To quantitate the volume of apoptotic particles or the number of glial cells, confocal stacks (five sections; total 7.5 *μ*m) were acquired from the neural cortex of stage-16 ventral nerve cords. To count DAC- or EVE-positive neurons, confocal stacks (15 sections; total 22.5 *μ*m) of three abdominal segments were acquired. To count CUT-positive neurons, confocal stacks (four sections; total 6 *μ*m) of three abdominal segments were acquired.

Live imaging was carried out following dechorionation of stage-15 embryos. LysoTracker (Molecular Probes, Leiden, The Netherlands) was injected at 2–3% of the egg volume and mounted in Halocarbon oil, as described.^52^

## Abbreviations

AP-1: activator protein 1
BSK: Basket
CA: constitutively active
CNS: central nervous system
DAC: Dachshund
dJNK: *Drosophila* c-Jun N-terminal kinase
DN: dominant negative
DRPR: Draper
EVE: even-skipped
HEP: Hemipterous
HID: Head involution detective
JNK: c-Jun N-terminal kinase
LT: LysoTracker
nGFP: nuclear green fluorescent protein
Puc: Puckered
REPO: Reverse Polarity
SIMU: Six Microns Under
wt: wild type
