# Supplementary material for: JNK pathway activation is able to synchronize neuronal death and glial phagocytosis in Drosophila
Source: Cell Death Dis. 2015 Feb 19;6(2):e1649–. doi: 10.1038/cddis.2015.27 (PMC4669801; doi:10.1038/cddis.2015.27)
Supplement: Supplementary Figure Legends [file cddis201527x1.doc]

**Online supplemental material**

**Supplemental figure S1. *puc* mutant embryos exhibit high volume of apoptotic particles outside the CNS.** (A-H) Projections from confocal stacks of the embryonic CNS at stage 16; ventral view. Bar 20 µm. (A-D) *puclacZ* heterozygous embryos as a control. (E-H) *puclacZ* homozygous mutant embryos. Anti-β-Gal (blue), CM1 (red) and anti-SIMU (green) antibodies. Mutant embryo shows accumulation of CM1-positive particles inside SIMU-labeled cells (macrophages) outside the CNS.

**Supplemental figure S2. The number of DAC positive neurons is similar in *puc* mutant and wild type embryos, at stage 14 of embryogenesis.** (A-C'') Projections from confocal stacks of young embryos; ventral view. Bar 20 µm. (A-C'') Embryos expressing *puclacZ* reporter stained with anti-β-Gal (red) and anti-SIMU (green) antibodies. Note the weak reporter activation at stage 13 (B') and stronger at stage 14 (C'). (D,E) Projections from confocal stacks of the embryonic CNS at early stage 14; ventral view. Bar 20 µm. Anti-DAC (red). (D) *puclacZ* heterozygous embryo as a control. (E) *puclacZ* homozygous mutant embryo. (F) Quantification of DAC-positive neurons at early stage 14. To count the number of DAC-positive neurons confocal stacks (15 sections; total 22.5 µm) were acquired. Columns represent mean total number of DAC-positive neurons within 3 segments of confocal stacks of the CNS, ± SEM, n=5-6 n.s. (not significant) p>0.05.

**Supplemental figure S3. *puc* mutant embryos exhibit no increase in the number of CUT-positive or EVE-positive neurons.** (A-B') Projections from confocal stacks of the embryonic CNS at stage 16; ventral view. Bar 20 µm. (A-A') Neurons labeled with anti-CUT (red). (B-B') Neurons labeled with anti-EVE (red). (A,B) *puclacZ* heterozygous embryos as a control. (A',B') *puclacZ* homozygous mutant embryos. (C-D) Quantification of different neuronal types. To count the number of EVE-positive neurons confocal stacks (15 sections; total 22.5 µm) were acquired. To count the number of CUT-positive neurons confocal stacks (4 sections; total 6 µm) were acquired from the neural cortex of stage 16 ventral nerve cords. Columns represent mean total number within 3 segments of confocal stacks of the CNS, ± SEM. (C) CUT-positive neurons. n=8. (D) EVE-positive neurons. n=8-9. (C-D) Asterisks indicate statistical significance versus control, as determined by Student’s t-test, *p<0.04, n.s. (not significant) p>0.05.

**Supplemental figure S4.** **dJNK pathway activation with *repoGal4::hepCA* in *simu* mutant** **embryos.** (A-B'') Projections from confocal stacks of the embryonic CNS at stage 16; ventral view. Bar 20 µm. Embryos stained with anti-JUN (red) and anti-REPO (green) antibodies. (A-A'') wild type embryo. (B-B'') *simu* mutant embryo expressing *repoGal4::hepCA*. Note stronger staining with anti-JUN in glial cells of *simu;repoGal4::hepCA* embryos indicating activation of the dJNK pathway.

**Supplemental figure S5. Induced neuronal apoptosis does not activate dJNK pathway in embryonic or larval neurons and glia.** (A-C'') Projections from confocal stacks of the CNS at embryonic stage 16, ventral view; *TRE-eGFP* reporter of dJNK activity in green, apoptotic cells in red (CM1). Bar 20 µm. *elavGal4::hid* (B-B'') and *elavGal4::reaper* (C-C'') embryos show a strong increase in the volume of CM1-positive particles compared to control (A-A''), but no change in GFP expression. (D-E'') 3^rd^ instar larval brains; *TRE-eGFP* reporter in green, apoptotic cells in red (CM1).
