# Supplementary figures and images for: JNK pathway activation is able to synchronize neuronal death and glial phagocytosis in Drosophila
Source: Cell Death Dis. 2015 Feb 19;6(2):e1649–. doi: 10.1038/cddis.2015.27 (PMC4669801; doi:10.1038/cddis.2015.27)

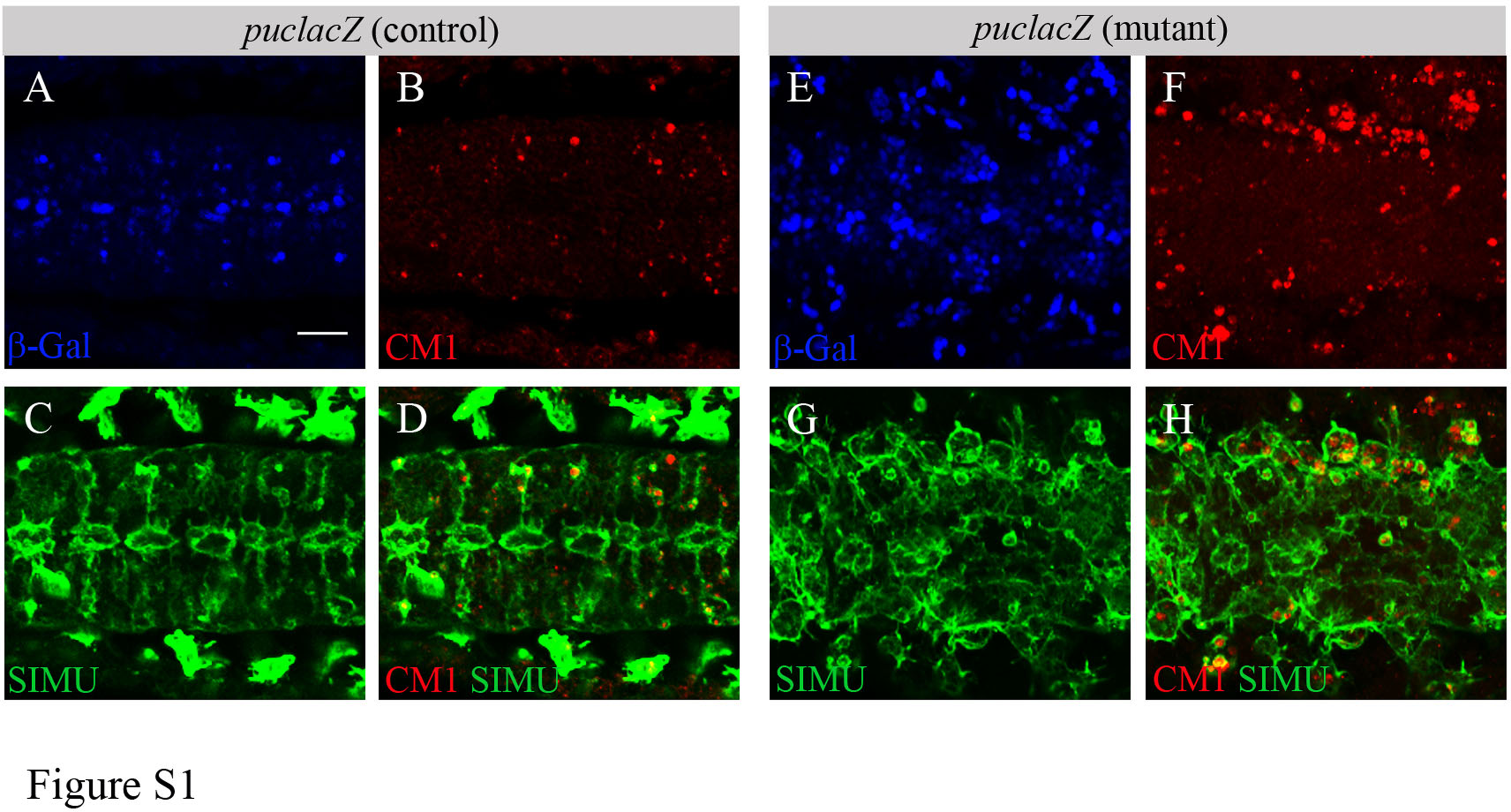

Supplement: Supplementary Figure 1 [file cddis201527x2.tif]

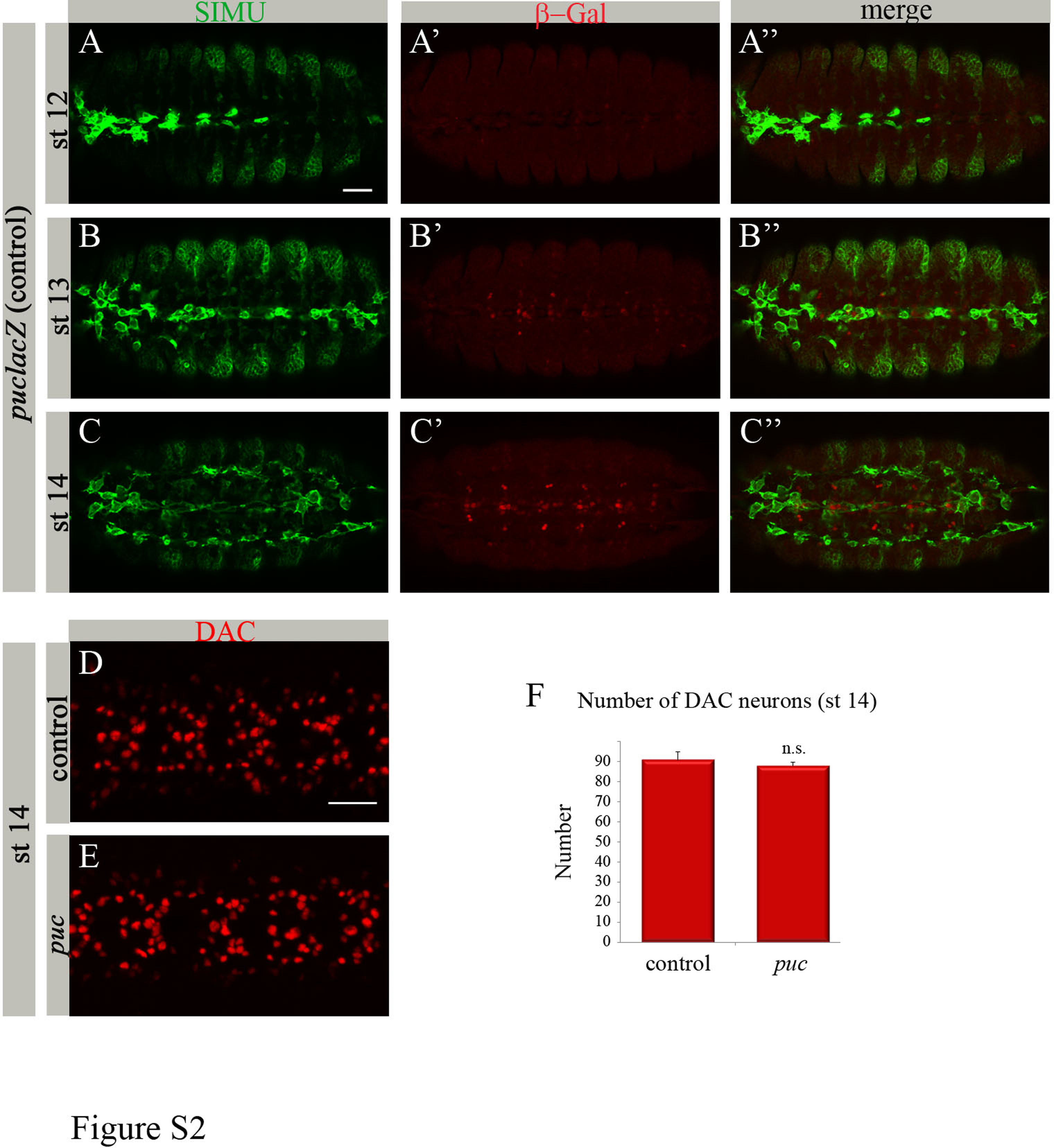

Supplement: Supplementary Figure 2 [file cddis201527x3.tif]

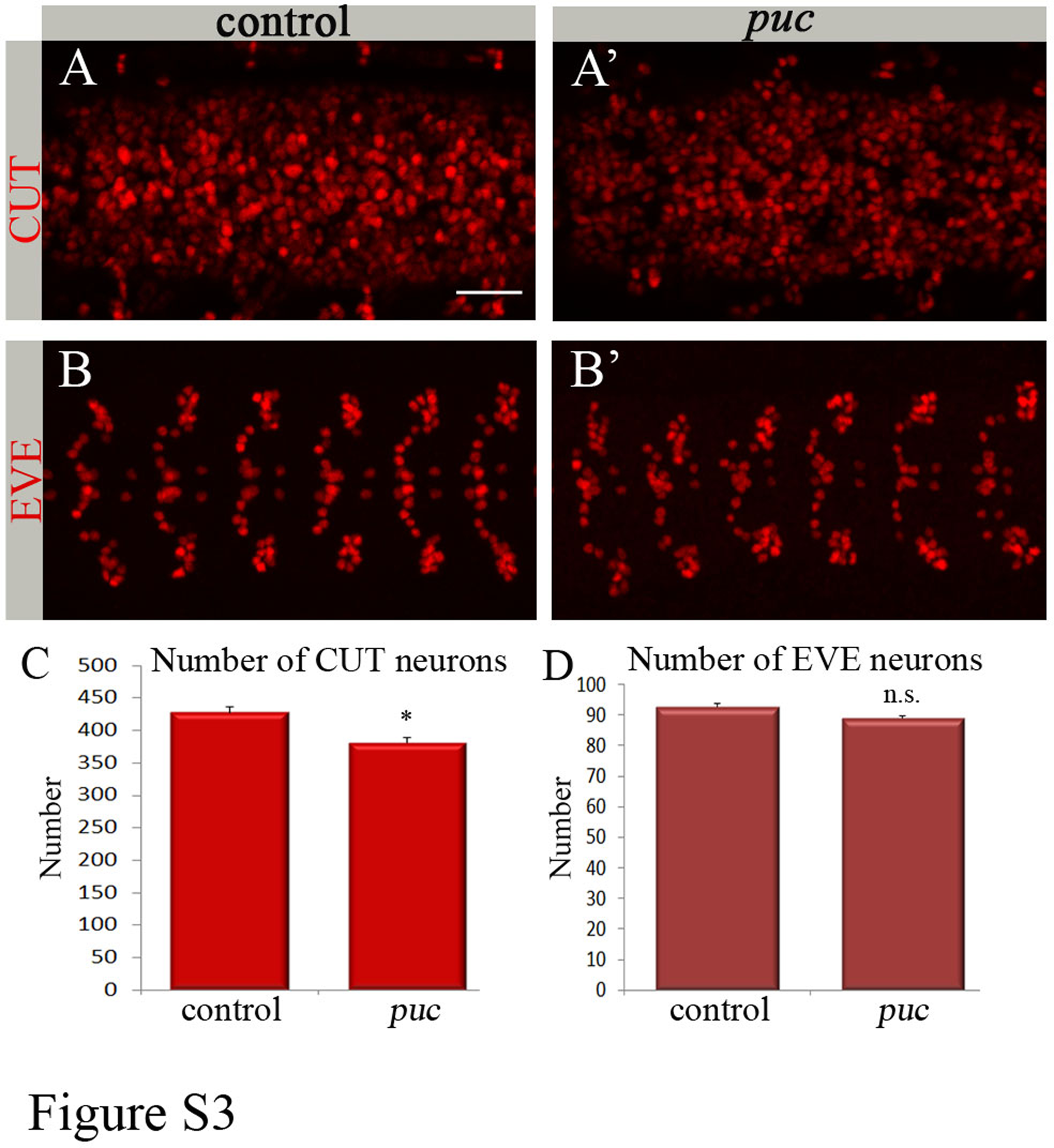

Supplement: Supplementary Figure 3 [file cddis201527x4.tif]

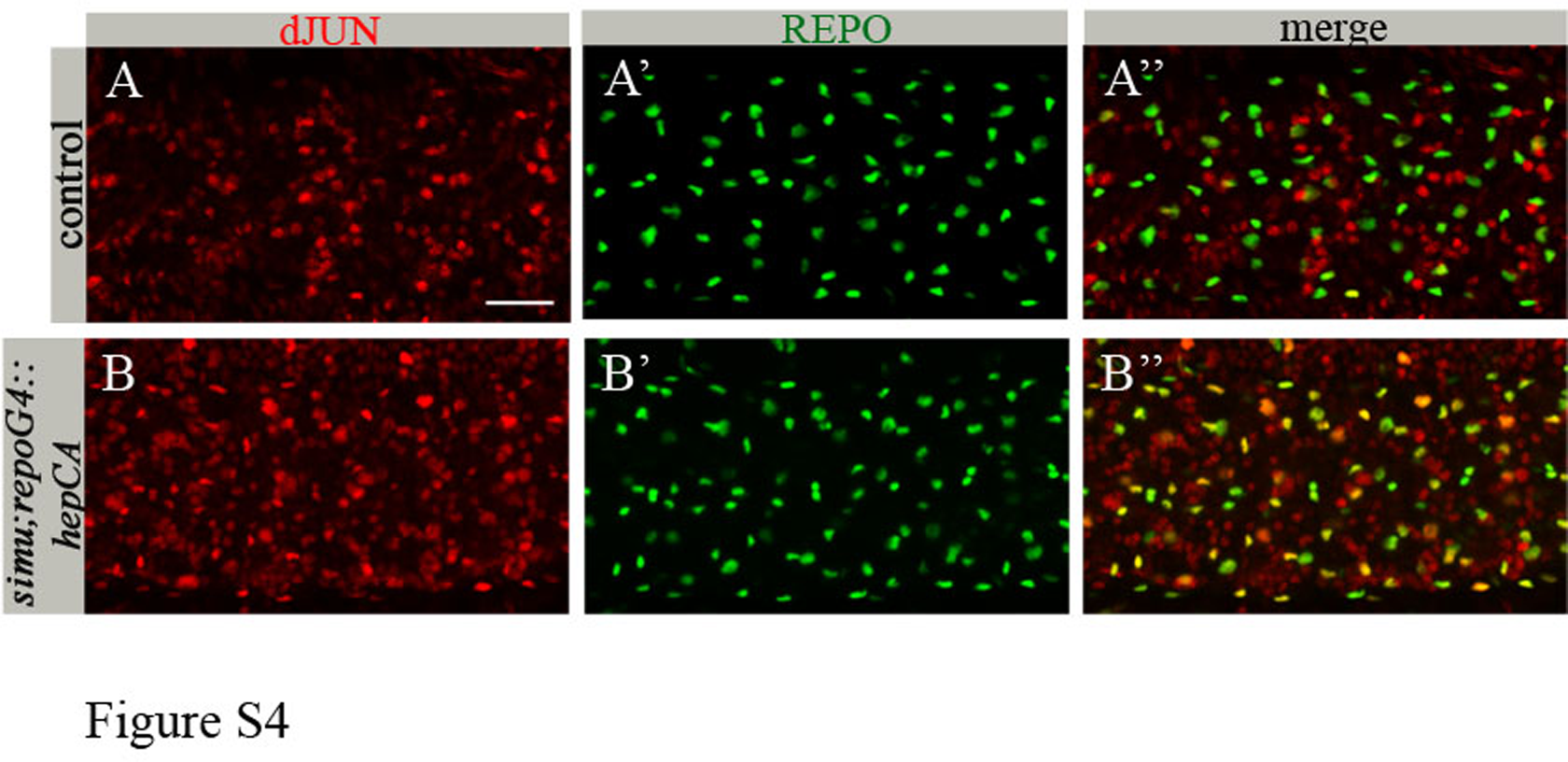

Supplement: Supplementary Figure 4 [file cddis201527x5.tif]

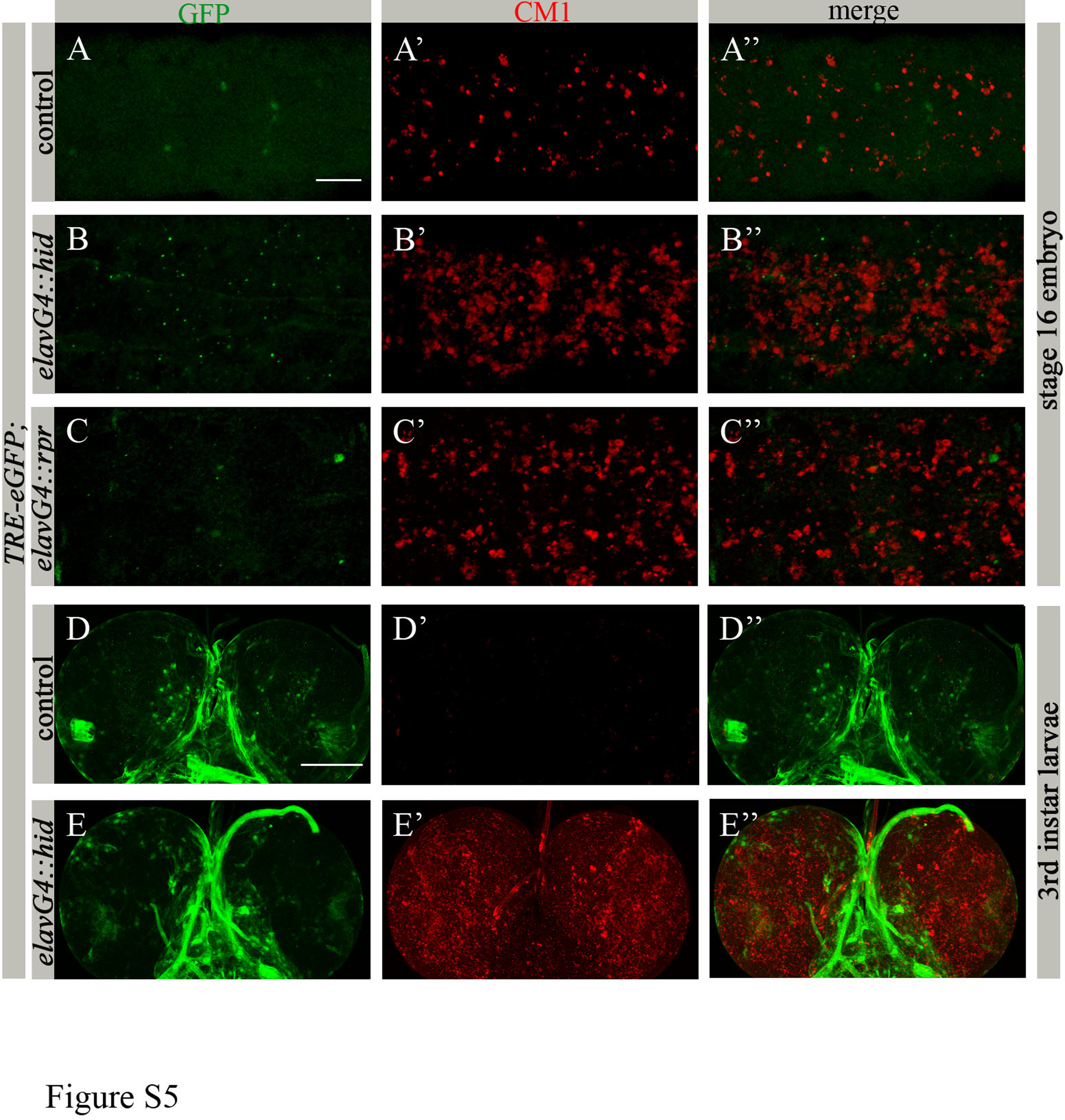

Supplement: Supplementary Figure 5 [file cddis201527x6.tif]
